# Semi-Automatic Analysis of Specific Electroencephalographic Patterns during NREM2 Sleep in a Pediatric Population after SARS-CoV-2 Infection

**DOI:** 10.3390/jpm14020152

**Published:** 2024-01-30

**Authors:** Paolo Di Bella, Anna Gaia Attardi, Ambra Butera, Arianna Mancini, Nunzia Calabrò, Elisa Giuseppa Lo Re, Giuseppe Trimarchi, Antonio Gennaro Nicotera, Gabriella Di Rosa, Daniela Lo Giudice

**Affiliations:** 1 Unit of Child Neurology and Psychiatry, “G. Martino” University Hospital, 98125 Messina, Italy; pdibella@unime.it (P.D.B.); a.gaia.attardi@gmail.com (A.G.A.); butera.ambra@gmail.com (A.B.); arianna.mancini89.am@gmail.com (A.M.); nunzia.calabro@yahoo.it (N.C.); elisag.lore@libero.it (E.G.L.R.); 2 SIR—Faculty of Medicine and Surgery, University of Messina, 98125 Messina, Italy; giuseppe.trimarchi@unime.it; 3 Department of Biomedical and Dental Sciences and Morphofunctional Imaging, University of Messina, 98125 Messina, Italy; gdirosa@unime.it (G.D.R.); dlogiudice@unime.it (D.L.G.)

**Keywords:** EEG, sleep spindles, slow spindles, fast spindles, post COVID19 condition, SARS-CoV-2, pediatric, NREM2 sleep

## Abstract

The post-COVID-19 condition is defined by the World Health Organization as the persistence of symptoms or development of new symptoms three months after the initial SARS-CoV-2 infection, lasting for at least two months without a clear explanation. Neuropsychiatric disorders associated with this condition include asthenia, memory and concentration problems, and sleep disturbances. Our study aims to investigate sleep patterns following SARS-CoV-2 infection using EEG findings and a sleep quality questionnaire completed by parents (Sleep Disturbance Scale for Children—SDSC). Notably, our investigation is based on a convenience sample. The patients in our sample, aged 1 to 14 years, are not currently taking any medications; rather, they are undergoing follow-up assessments at the Child Neuropsychiatry department of the University Hospital of Messina for neurodevelopmental evaluations. Specifically, we are analyzing amplitude and power spectrum data in the first five minutes of NREM2 sleep, calculated from EEG recordings obtained via bipolar leads within three months after the onset of the disease. These results will be compared with controls performed on the same subjects in the six months preceding the infection. The focus of the study was sleep spindles, which are generated by the thalamocortical systems and play a role in sleep modulation, memory, and learning. Preliminary analysis suggests a predominant increase in the slow component of the spindles in the right-frontal lead.

## 1. Introduction

The emergence of the novel severe acute respiratory syndrome coronavirus 2 (SARS-CoV-2) in 2019 precipitated the global COVID-19 pandemic [1]. Initially identified in China, the virus was rapidly disseminated worldwide, leading to a substantial loss of lives. Consequently, on 11 March 2020, the World Health Organization (WHO) declared COVID-19 a pandemic [2].

COVID-19 is a highly contagious viral disease that primarily affects the respiratory, gastrointestinal, and neurological systems, manifesting in varying degrees of severity [1,3].

It is important to note that children seldom develop severe symptoms of COVID-19. More commonly, they exhibit mild symptoms or remain asymptomatic. However, complications can arise, including a condition known as multisystem inflammatory syndrome in children (MIS-C). MIS-C, a severe complication, can manifest 2 to 6 weeks after the initial infection and may share diagnostic criteria with Kawasaki disease (KD) [4].

Another condition associated with the primary infection is “Post-COVID-19” (also referred to as long COVID), characterized by persistent symptoms. The WHO defines post-COVID-19 as “the persistence or development of new symptoms three months after the initial SARS-CoV-2 infection, lasting for at least two months with no other explanation in individuals with a history of probable or confirmed infection” [2].

These symptoms may arise following the initial resolution of the acute COVID-19 episode or persist following the initial infection. They include recurrent fever, rash, flu-like symptoms (asthenia and muscle aches), cardio-respiratory symptoms (shortness of breath, palpitations, chest pain, and persistent cough), gastrointestinal issues (diarrhea and abdominal pain), and neuropsychiatric disorders (headache, anosmia, memory and concentration problems, cognitive impairment, and sleep and mood disorders) [1,2].

SARS-CoV-2 exerts its neurotropic effects through multiple mechanisms, potentially leading to or exacerbating neurological dysfunction. This dysfunction may result from various factors, such as direct viral encephalitis, neuroinflammation (resulting in blood-brain barrier disruption), hypoxia, and cerebrovascular disease. Several studies in the literature have indicated the presence of brain lesions in anatomical samples from COVID-19 patients [5,6,7].

Using polysomnography (PSG), some authors have shown that EEG changes during sleep are common in COVID-19 survivors. Among the recorded alterations were an increased presence of alpha waves during sleep and increased REM density [8].

Data from the literature emphasize the association between COVID-19 and sleep disturbances. These abnormal waves suggest that this neurotropic virus may have effects similar to other neuropsychiatric disorders that influence physiological sleep activity [8]. For example, insomnia and obstructive sleep apnea have been associated with alpha intrusion and increased spindles. Conversely, major depression and schizophrenia (in severe forms at increased risk of suicidality) have been linked to increased REM sleep density [9,10,11].

These effects have been observed only in short-term follow-up studies, making it only possible to define the long-term effects by conducting serial polysomnography in patients with COVID sequelae [8].

In this context, we proposed a study that combines EEG and the Sleep Disturbance Scale for Children (SDSC), filled out by caregivers, to evaluate the effects of COVID on the electrical brain activity of children diagnosed with neurodevelopmental disorders.

The Sleep Disturbance Scale for Children (SDSC) is an easy-to-complete questionnaire for the collection of data on children’s sleep behavior. It consists of 26 caregiver-focused questions that assess sleep habits in children, exploring various sleep characteristics throughout the night, as well as at bedtime and wake-up time [11].

In our research, data related to the amplitude and power of sleep spindles, specifically the power of fast and slow spindles, calculated based on EEG recordings conducted within 3 months of the disease onset, are compared with controls performed on the same subjects in the 6 months before the infection. Analyses conducted so far reveal a significant increase in the slow component of the spindles on the right-frontal lead [8,11].

## 2. Material and Methods

The study utilized data from 24 children aged between 2 and 14 years under the care of the UOC of Child Neuropsychiatry at Policlinico “G. Martino” in Messina, Italy. These children underwent clinical examinations and electroencephalograms (EEGs) as part of neurodevelopmental assessments within six months prior to testing positive for COVID-19 via the PCR real-time molecular test. None of the children displayed significant clinical signs, EEG abnormalities, or unusual graph elements.

All children in the study tested positive for COVID-19, and their infections were confirmed through the PCR real-time molecular test. As reported by their parents, none of the children required hospitalization for COVID-19.

Post-COVID Symptoms: Despite not requiring hospitalization, the parents reported that all the children displayed lingering symptoms consistent with the post-COVID-19 condition. Similar to those seen in adults, these symptoms included various manifestations affecting different systems (e.g., cardiovascular, respiratory, muscular, gastrointestinal, and neurological). Notable post-COVID neurological manifestations reported by the parents included mental fog, concentration difficulties, sleep disturbances, dizziness, irritability, mood changes, headache, memory loss, loss of smell, loss of taste, and night sweats [12].

Follow-Up Evaluation: All children underwent a new evaluation and EEG recording once they had tested negative for COVID-19, and this was performed within a time frame not exceeding three months from the date of the initial positive COVID-19 test.

Throughout the study period, both before and after COVID-19 infection, none of the children received any form of pharmacological therapy.

Only right-handed patients were included in the sample when it was possible to distinguish their handedness.

The EEG recordings of the patients were examined after obtaining consent from the parents or guardians of the children involved. These EEG recordings were performed after hypnic deprivation of the children. The children were allowed to fall asleep at midnight and woke up at 5 AM. Sleep deprivation in young children was carefully managed with engaging activities suggested by the parents. Additionally, they were instructed not to consume stimulating substances such as caffeine or theine in the 24 h prior to the recording session. Significant rebound sleep effects were not observed during the study.

It is important to note that our study design relies on a convenience sample rather than a prospectively enrolled cohort.

### 2.1. Assessment of Sleep Quality

The SDSC was validated by Bruni et al. in 1996 as a tool to detect sleep disorders in children; this questionnaire consists of 26 points, which can be scored from 1 to 5, and is addressed to the child’s parent who evaluates the child’s sleep characteristics over the last six months. While for the first two items, the scores from 1 to 5 evaluate the hours of sleep and the hours needed to fall asleep, for the other items, which refer to the behaviors adopted by the child, the values from 1 to 5 indicate the frequency with which this behavior occurs. Among the sleep disorders, the questionnaire mainly considers difficulty falling asleep and maintaining sleep, breathing problems, hyperarousal disorders, sleep-wake transition alterations, daytime sleepiness, and hyperhidrosis. It was initially developed for children between the ages of 6 and 15 [13]. However, it was also validated for those in the Italian population aged between 3 and 36 months [11,14].

We considered the questionnaire clinically significant if the total score obtained by summing the items corresponded to a T score equal to or greater than 70 [15].

### 2.2. Evaluation of EEG Parameter

The examination sessions consisted of a video-EEG recording of wakefulness and subsequent falling asleep and onset of SNREM phase 2, lasting at least 5 min.

An EEG system (Galileo, EBNeuro) was used to record and sample at 256 Hz. The EEG signals were acquired using pre-wired headphones suitable for the patient’s age and cranial circumference, with a minimum of 10 exploratory electrodes positioned on the leads: Fp1, Fp2, F3, F4, C3, C4, T3, T4, O1, and O2 as indicated by the SI. During re-reading, trace visualization was performed using a low-pass filter with a value of 70 Hz and a high-pass filter with a value of 0.3 Hz. In addition, a 50 Hz notch filter was applied.

In order to provide a homogeneous sampling of the N2 phase, we considered only the first 5 min and calculated the average. This is the minimum time for all observations conducted on the patients.

All intervals with evident artefactual activity were eliminated from the analysis using a selection (carried out by two referring physicians who were experts in reading the electroencephalographic tracings of children of developmental age), which included the absence of eye movements and/or contamination of other physiological sleep activities.

**Figure 1:**
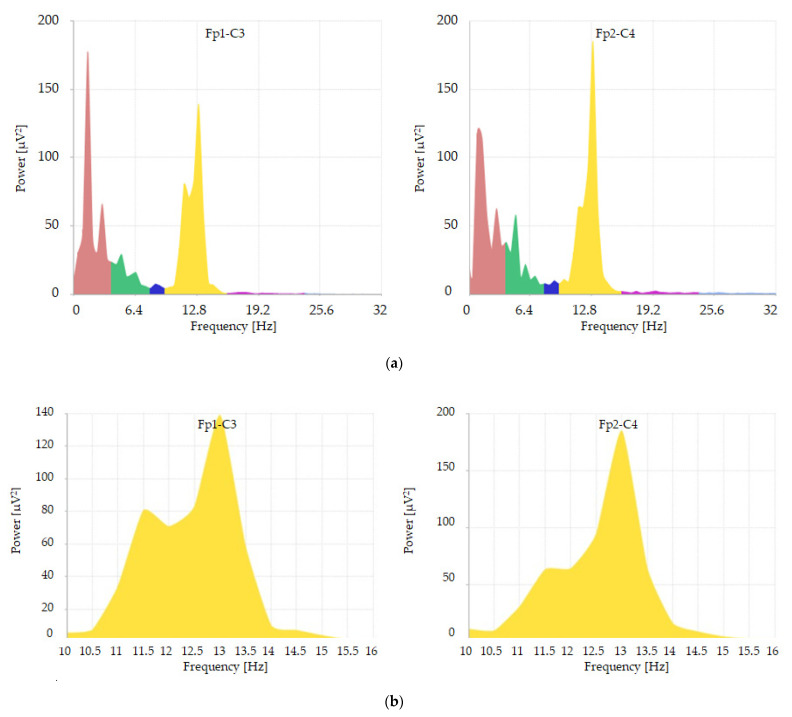
Example of a spectral analysis distribution, depicting powers expressed in microvolts^2^, derived from Fp1-C3 and Fp2-C4 leads of a patient enrolled in our study. The distribution spans a frequency range from 0 to 32 Hz (**a**), with a detailed view of the frequency range from 10 to 16 Hz provided in (**b**).

**Figure 2:**
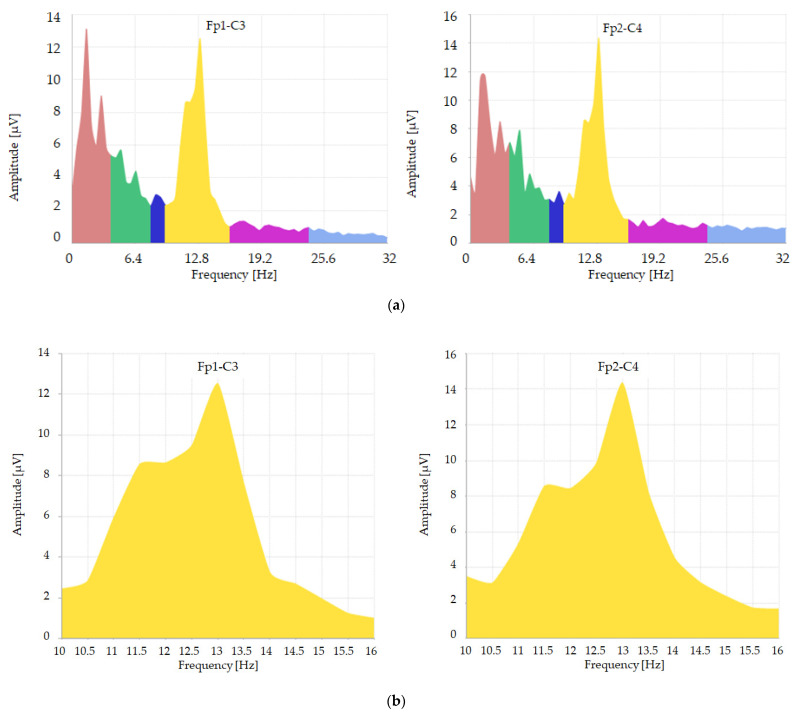
Example of a spectral analysis distribution, depicting amplitude expressed in microvolts, derived from Fp1-C3 and Fp2-C4 leads of a patient enrolled in our study. The distribution spans a frequency range from 0 to 32 Hz (**a**), with a detailed view of the frequency range from 10 to 16 Hz provided in (**b**).

From the EEG data intervals of interest, three-second traces containing the hypnosis figures under study were then exported and analyzed in terms of power (Figure 1) and amplitude (Figure 2) using the Galileo System previously used for the recordings.

Referring to (a) graphs of these figures (1 and 2): the abscissa values referring to “frequency Hz” are colored according to the subdivision of the different bands: delta 0–4 Hz as PINK; theta 4–8 Hz as GREEN; alpha 8–12 Hz as BLUE; beta1 12–24 Hz as PURPLE; beta2 24–32 Hz as LIGHT BLUE. The YELLOW-colored component (better studied in graphs (b) of these figures (1 and 2)) corresponds to a frequency range with values between 10 Hz and 16 Hz; it incorporates final part of the alpha band (10–12 Kz) and initial part of the beta1 band (12–16 Hz). This subdivision was necessary for extrapolation of the raw data subsequently subjected to statistical analysis.

The data were obtained from the following leads: right-frontal polar (Fp2/F R); left-frontal polar (Fp1/F L); right-central (C4/C R), and left-central (C3/C L).

A frequency range of 10 Hz to 16 Hz was used for the evaluation, with a further subdivision involving separate analysis for the slow component (10–13 Hz) and the fast component (13–16 Hz).

For the analysis of sigma activity, N2 epochs subdivided into 3-s segments using the fast Fourier transform (FFT) algorithm provided power spectra relative to:

- Absolute-power fullband;
- Amplitude full band;
- Medium-frequency full band;
- Median-frequency full band;
- Slow-component absolute power;
- Slow-component medium frequency;
- Fast-component absolute power;
- Fast-component medium frequency.

### 2.3. Statistical Data Analysis

Descriptive and inferential analyses were performed using R software (rel. 4.2.0). Continuous data were presented using the median and IQR, and categorical parameters using absolute frequency and percentage.

All quantitative parameters were tested for normality using the Shapiro–Wilk test. Only the age parameter was discriminated by gender and sleep changes, and the significance was tested by Student’s *t*-test or Mann–Whitney’s U-test when necessary.

Associations between all categorical parameters (gender, sleep changes, and diagnosis) were assayed by the chi-square test.

Pre and post-COVID-19 spectral analysis data were assayed by paired t-test or Wilcoxon sign test when appropriate.

Values less than *p* = 0.05 were considered for statistical significance.

## 3. Results

### 3.1. Sample Characteristics

Our sample consisted of 24 patients aged 2 to 14 years (median 5; IQR 4): 16 males and nine females. There is no statistically significant relationship between gender (median females 5, IQR 4; median males 5, IQR 4) and age (*p* value, 0.953), as expected from a homogeneous sample.

### 3.2. SDSC Sleep Disturbance Scale for Children

The caregivers, typically parents, completed the SDSC questionnaire regarding their child’s sleep characteristics within three months of the child’s positive PCR Real-Time molecular test for SARS-CoV-2. Of the 24 completed questionnaires, 8 showed clinically significant results for sleep disorders (T score equal to or greater than 70). The questionnaires revealed that sleep was disturbed in 1/3 of the patients. The statistical data for the relationships between age and sleep changes (no (<70) median 4.50, IQR 3.50; yes (>70) median 6.00, IQR 5.50; *p* value 0.452) and that between gender and sleep changes (*p* value 0.371) (Table 1) showed no statistically significant correlations. Other external variables could have influenced sleep disturbances, among which COVID-19 could not be excluded.

### 3.3. Analysis of EEG Parameters Compared

Due to the substantial variability in the collected data, differences between the parameters derived from pre- and post-COVID-19 records were frequently found to be statistically insignificant. This variability can also be attributed to the types of parameters used. The results of the conducted analyses are summarized in Table 2. Statistically significant differences were observed for the following variables: right-frontal sigma power, right-frontal sigma mean, central-left sigma median, and right-frontal sigma slow-spindle power. The significance of these results is underlined by the increased uniformity of the parameters among all patients. The statistically significant differences, particularly those related to the power of the right-frontal leads, are particularly noteworthy. These findings are especially true in the case of the slow component of the spindles: frontal-right sigma power (*p*-value 0.035), which indicates an increase in power in the months following COVID-19 (median before 56.37 vs. median after 57.39; IQR before 127.42 vs. IQR after 128.21), and right-frontal sigma slow-spindle power (*p*-value 0.014), showing an increase in slow spindle power in the months following COVID-19 (median before 31.63 vs. median after 35.06; IQR before 87.3 vs. IQR after 96.18).

## 4. Discussion

Our study aimed to investigate the characteristics of the physiological elements of the second stage of sleep, such as spindles, as a possible expression of changes in hypnic activity in children who had been infected with COVID-19 and presented symptomatology compatible with the post-COVID condition. For this reason, we compared the EEG tracings obtained prior to the infection with those obtained following infection. In addition, we asked the parents to complete a questionnaire on the children’s sleep during the last 6 months to assess the occurrence of perceived sleep disturbances.

A literature review shows that there are still few studies on this topic and even fewer in the pediatric field. Yılmaz et al. published a study on a pediatric population that had been mildly or moderately symptomatically infected with COVID-19 and underwent EEG testing with a subsequent comparison with healthy controls. Their study showed a general decrease in background activity and the presence of epileptic abnormalities in children who had contracted SARS-CoV-2 [16]. Goyal et al. performed an EEG study on patients with COVID-19 who had been discharged from the hospital 4–6 weeks earlier. This study showed the presence of the EEG patterns of alpha rhythm intrusions (present in 78% of patients) and alternating cyclic patterns (present in 59% of patients). In addition, an increase in spindles was evident in 16% of the cases. The author highlighted how the presence of these abnormalities means that COVID-19 has similarities to insomnia and depression, at least in the first period [17]. Appelt et al. observed in a study of 53 COVID-19 patients and 30 controls that the EEG tracings showed a reduction in activity in the frontal areas at 6–12 months post-infection with worsening scores on cognitive assessments [18]. In a study focusing more on sleep spindles, Rubega et al. showed that the frequency of slow spindles was reduced in patients hospitalized for COVID-19 compared to those of controls. They also found that, following infection, slow spindles were more posterior and fast spindles more anterior [19].

Given the small number of previous studies, it is crucial to further investigate the characteristics of sleep spindles and any changes to them in pediatric patients with COVID-19. These studies may also improve understanding of the mechanism by which SARS-CoV-2 affects the central nervous system. ACE 2, expressed by neuronal and glial cells, represents the ligand by which the virus enters the brain through circulation, cranial nerve I, and the olfactory bulb [18,20]. Several authors have observed that COVID-19 changes brain activity and connectivity and causes a pronounced reduction in the grey matter of the orbitofrontal cortex [6,18]. The EEG is a non-invasive technique to assess the effects of SARS-CoV-2 in the brain. SARS-CoV-2 preferentially affects the prefrontal cortex, basal ganglia, and hypothalamus, which are essential for the development of psychiatric symptoms and sleep regulation [5,17]. In our study, we focused on analyzing sleep spindles. These, generated by the interaction between the thalamus and cortex, are sleep stabilizers involved in synaptic plasticity, learning, and memory consolidation [21,22]. In young adults, symmetrical sleep spindles between the hemispheres have been correlated with improved task attention [7,9]. Furthermore, a link between spindles and attention has been identified through genetic association studies in neurodevelopmental disorders such as autism spectrum and ADHD, both characterized by late falling asleep and frequent awakening. Several risk genes for these disorders are strongly expressed in the TRN (thalamic reticular nucleus), a collection of locally projecting neurons located between the thalamus and the cortex, providing potential anatomical substrates for spindle-like rhythms in all dorsal thalamic nuclei [9,23].

During our research, it was not possible to determine the frequency of the spindles due to the mode and duration of the recordings, for this reason we chose to analyze only the first few minutes of the NREM2 phase. An increase in the power of the spindles in the right-frontal lead, especially the power of the slow spindles, was observed when comparing the parameters obtained in our study. The transition point from slow to fast spindles is around 13 Hz, and while the fast spindles appear more posterior, the slow spindles appear more anterior [9]. The slow component of the spindles originates in the frontal gyrus and is associated with verbal memory, which in turn is closely linked to attention and learning [19,24,25]. Furthermore, the speed of information processing in children, defined as the time it takes to solve a given problem, has been positively correlated with the density of slow spindles by some authors. In contrast, no correlation has been observed with fast spindles, which could indicate a relationship between slow spindles and cognitive development in children [21,26].

The results of our research seem even more interesting when correlated with the neurological symptoms reported by our patients, which are compatible with the post-COVID-19 condition (mental fog, concentration difficulties, sleep disturbances, dizziness, irritability and mood changes, headaches, memory loss, loss of smell, loss of taste, and night sweats) [12].

The SDSC (Sleep Disturbance Scale for Children) questionnaire was positive (total score > 70) in 8 out of 24 patients: in 1/3 of the patients, the parents reported sleep disturbances. This result confirms the finding in the literature of sleep disturbances in the pediatric post-COVID condition) [12]. However, it should also be correlated with the stress of the restrictive measures during the pandemic, especially for the pediatric population. In addition, this questionnaire was only completed by parents and indicates sleep disturbances with several possible causes. However, it must be stressed that our sample’s age influenced the choice of questionnaire.

Examples of other unexamined variables that could have influenced the results include pre-existing underdiagnosed sleep disorders, psychosocial factors, parental psychological well-being, and changes in daily routine. In the future, possibly with a larger sample size, we would like to test whether there is a correspondence between the patients for whom the questionnaire is positive and those for whom the statistically significant differences found at the level of sleep spindles are most pronounced.

The small size of the sample, consisting of patients within a rather wide developmental age range (2–14 years), and the lack of control cases are major limitations of the study. Because of the great variability in the characteristics of the sleep spindle over the course of development in each individual, a short time interval was necessary between the pre-infection and post-infection recordings; for this reason, owing to the numerous criteria for inclusion (age between 2–14 years, pre-infection electroencephalogram within the normal range within 6 months, post-infection electroencephalogram within 3 months, absence of drug therapy, and symptoms compatible with the post-COVID-19 condition), we isolated only 24 patients who presented the required characteristics. For the same reasons, it was difficult to identify controls.

Another limitation of the study is the lack of a homogeneous testological assessment for the cognitive and attentional abilities of the children studied to accompany the electroencephalogram; given the expected increase in COVID-19 cases in the coming months, this could be the starting point for a future study design.

Moreover, there needed to be a control group in our study. This limitation was addressed by emphasizing the exploratory nature of the study and the intention for future investigations that incorporate control cases to be conducted. We recognize that the lack of a control group may impact our analyses’ comprehensiveness and the results’ generalizability.

One of the strengths of the study is that it examines an area that is still little explored, especially in the pediatric population, with all the possible social and ethical implications. In particular, the finding of an increase in the power of the spindles of the right-frontal lead, and especially the slow component, in pediatric patients with post-COVID-19 syndrome confirms the usefulness of carrying out electroencephalographic checks in these patients. At the same time, it allows us to identify sleep spindles as possible diagnostic and therapeutic targets in the clinical management of the consequences of the current pandemic.

## 5. Conclusions

Our study showed a significant difference in spindle power, mainly slow-spindle power, in the right frontal lobe in the electroencephalographic recordings of pediatric patients with COVID-19 compared to that shown in recordings taken before infection. In one-third of the cases, the questionnaire completed by the parents of the affected children was positive for sleep disturbance. This result confirms the importance of studying sleep, particularly sleep spindles, after SARS-CoV-2 infection and the potential link between spindle characteristics and the neurocognitive symptoms of the post-COVID-19 condition.

Therefore, our study suggests several promising paths for further exploration. Longitudinal studies emerged as a critical avenue, as they would offer the potential to uncover whether the observed changes in sleep spindles endure over time or represent transient phenomena. Such an investigation is imperative for gaining insights into the lasting implications of neurocognitive symptoms post-COVID-19.

Additionally, to address the limitations of our current study, including control cases in future research endeavors is a vital consideration. By doing so, we can deepen our analysis, fortify the applicability of our findings, and establish a basis for comparing the alterations in sleep spindles between the affected and unaffected groups.

Another aspect deserving attention involves conducting a more homogeneous testological assessment. Future studies should comprehensively evaluate cognitive and attentional capacities in the studied children, complementing the electroencephalogram recordings. This approach would provide a nuanced understanding of the intricate interplay between changes in sleep spindles and cognitive functions.

Furthermore, the potential diagnostic significance of sleep spindles opens the door to exploring therapeutic interventions. Future research endeavors could explore strategies targeting sleep in pediatric patients grappling with post-COVID-19 syndrome. Understanding the modifiability of sleep spindles could pave the way for interventions to improve neurocognitive outcomes in this population.

## Figures and Tables

**Table 1:** The relationship between gender and sleep changes is not statistically significant (chi square: 0.800; *p* = 0.371).

Sleep Changes	All Patients	Females	Males
All Patients	24	9	15
No (<70)	16	5	11
	66.7%	55.6%	73.3%
Yes (>70)	8	4	4
	33.3%	44.4%	26.7%

**Table 2:** Median and IQR of the data of all patients (n = 24) before and after infection are reported in the table. When *p* value < 0.05 there is a statistically significant difference between the values examined.

Sigma	Median Ante	IQR Ante	Median Post	IQR Post	*p* Value
F L Power (μV^2^)	59.73	48.19	61.16	55.59	0.34
F R Power (μV^2^)	56.37	127.42	57.79	128.21	0.035
C L Power (μV^2^)	197.19	78.72	200.17	88.13	0.607
C R Power (μV^2^)	156.55	129.18	162.82	102.07	0.909
F L Amp. (μV)	17.78	7.27	18.45	8.16	0.819
F R Amp. (μV)	17.5	13.91	18.04	14.06	0.092
C L Amp. (μV)	32.83	7.48	32.19	5.5	0.627
C R Amp. (μV)	29.11	10.07	29.11	8.71	0.932
F L Mean (Hz)	12	0	12	0.5	0.48
F R Mean (Hz)	12	1	12	0.5	0.046
C L Mean (Hz)	12	1	12	0.5	0.317
C R Mean (Hz)	12.5	1	12	1	0.157
F L Median (Hz)	12.3	0.58	12.23	0.63	0.378
F R Median (Hz)	12.31	0.79	12.3	0.7	0.223
C L Median (Hz)	12.41	0.46	12.31	0.5	0.014
C R Median (Hz)	12.42	1	12.15	0.9	0.051
F L Sl power (μV^2^)	40.65	34.31	44.01	37.19	0.429
F R Sl power (μV^2^)	31.63	87.3	35.06	96.18	0.014
C L Sl power (μV^2^)	103.30	85.53	95.24	89.93	0.909
C R Sl power (μV^2^)	63.51	85.88	70.03	89.83	0.331
F L Fa power (μV^2^)	19.18	20.88	20.92	17.95	0.123
F R Fa power (μV^2^)	22.10	17.03	21.46	14.9	0.864
C L Fa power (μV^2^)	81.63	66.84	67.87	60.09	0.097
C R Fa power (μV^2^)	46.05	70.67	45.85	70	0.278
F L Sl mean (Hz)	11	1	11	0.50	0.564
F R Sl mean (Hz)	11	1	11	1	*
C L Sl mean (Hz)	11	0.50	11	1	0.317
C R Sl mean (Hz)	11	1	11	0.50	0.3017
F L Fa mean (Hz)	14	0.50	14	0.50	*
F R Fa mean (Hz)	14	1	14	1	*
C L Fa mean (Hz)	14	1	13.5	1	0.564
C R Fa mean (Hz)	14	1	14	1	0.317

Legend: Amp. = amplitude; F = frontal polar; C = central; R = right; L = left; Sl = slow spindles; Fa = fast spindles; * not calculable.

## Data Availability

The data were obtained from electroencephalographic recordings kept in the electronic archive of the electroneurophysiology laboratory of the Division of Child Neurology and Psychiatry, “G. Martino” University Hospital, 98125 Messina, Italy.
